# Potential Development of Vitrified Immature Human Oocytes: Influence of the Culture Medium and the Timing of Vitrification

**DOI:** 10.3390/ijms24010417

**Published:** 2022-12-27

**Authors:** Irene Peinado, Isabel Moya, Laura García-Valverde, Raquel Francés, Rosana Ribes, Patrocinio Polo, María José Gómez-Torres, Ana Monzó

**Affiliations:** 1 Assisted Human Reproduction, La Fe University and Polytechnic Hospital, 46026 Valencia, Spain; 2 Biotechnology Department, Alicante University, 03690 Alicante, Spain; 3 Energy and Memory, Brain Plasticity Unit, CNRS, ESPCI Paris, PSL Research University, 75005 Paris, France; 4 Cátedra Human Fertility, Alicante University, 03690 Alicante, Spain

**Keywords:** germinal vesicle oocyte, in vitro maturation, cryopreservation, embryo development, vitrification, survival

## Abstract

How does the in vitro maturation (IVM) medium and the vitrification procedure affect the survival of germinal vesicle (GV) oocytes obtained from stimulated cycles and their development to the blastocyst stage? In total, 1085 GV human oocytes were obtained after women underwent a cycle of controlled ovarian stimulation, and these oocytes were subjected to IVM before or after their vitrification. IVM was carried out in two commercial culture media not specifically designed for maturation. MII oocytes were then activated and embryo development until day 6 was evaluated. According to the results, a higher percentage of oocytes reach the MII stage if they are vitrified before they undergo IVM. Nevertheless, the medium used and the sample size determine whether these differences become significant or not. Similar survival rates and development to blastocysts were observed in all the conditions studied.

## 1. Introduction

The proper combination of two assisted reproductive techniques (ARTs), specifically oocyte vitrification (OV) and in vitro maturation (IVM), represents an interesting strategy and improvements to these approaches may enhance their output.

In recent years, many children have been born from vitrified [1,2,3,4], IVM [5,6,7,8,9,10], or IVM and vitrified oocytes [11,12]. While OV is considered to be a consolidated technique that produces good results in ART laboratories, IVM still presents deficient and poorly reproducible results, a procedure that remains in an experimental phase.

OV is associated with high survival rates (SRs), both when oocytes are vitrified at the germinal vesicle (GV) or metaphase II (MII) stage [13,14,15,16]. Nevertheless, the maturation, fertilization, and development rates of GV after OV remains controversial [17,18,19]. In theory, the DNA of GV should be more resistant to cryodamage as it is highly compact at this meiotic state and protected by the nuclear membrane. 

The clinical implementation of IVM has progressed slowly due to technical problems associated with this procedure and one of the main stumbling blocks has been the choice of an adequate culture medium (CM) for maturation. Different media for oocyte maturation have been studied over the years, such as human tubal fluid (HTF [20]), cell culture medium (199, IVF [21,22]), medium for the culture to blastocyst [6,22,23,24], or specific commercial media for IVM [9]. Maturation rates have improved over time and although some of these culture media have achieved good maturation rates, the results have not been sufficiently reproducible to establish them as the reference medium of choice for IVM.

Recent studies in this field have focused on improving maturation rates and on defining the best meiotic stage for OV. The quality of the mature oocytes obtained after the combination of these ARTs was evaluated by analyzing them by electron microscopy [25,26], confocal microscopy [27,28,29], epigenetics [30,31,32,33], or through their subsequent embryonic development [6,23]. 

Therefore, it is clearly important to develop and optimize a protocol that achieves the best vitrification/maturation of GV, which will greatly improve the success of in vitro fertilization (IVF) cycles. As such, in this study, oocyte survival and embryonic development was evaluated as a means to assess the efficiency of IVM before or after vitrification of human immature oocytes in two different culture media: (1) gamete and early-stage embryo culture medium; and (2) embryo to blastocyst culture medium. The goal was to establish a protocol in which both these events were optimized so as to enhance the efficiency of IVF procedures.

Our results showed comparable vitrification survival and blastocyst development rates regardless of the medium used or the maturation stage of the oocytes. However, this study exhibited higher maturation rates whether oocytes were cryopreserved before IVM and/or when they were cultivated in the commercial supplemented maturation medium (human menopausal gonadotropin (hMG) + synthetic serum substitute (SSS)) designed to reach the blastocyst stage. 

## 2. Results

### 2.1. Survival Rate (SR)

The previous maturation state of the oocytes did neither significantly influence the SR of the oocytes after warming (GV-Vit 84.3% (419/497), MII-Vit 85.7% (234/273)), nor was the SR influenced by the specific medium used for maturation [CM1 86.7% (98/113), CM2 85.0% (136/160)].

The comparison of the effect of the IVM medium on SR was only assessed for MII-Vit oocytes, as these were the only ones that may have seen their SR affected by the distinct maturation media used after vitrification.

### 2.2. Maturation Rate (MR)

The MR was used to assess whether prior vitrification of the oocytes affected this IVM process and we found that a significantly higher proportion of the warmed oocytes resumed meiosis (GVBD) than the fresh oocytes (GV vitrified 83.7% (349/417), GV fresh 73.0% (374/512); *p* < 0.0001). This was also witnessed when the MR was evaluated after 24 h (GV vitrified 59.2% (247/417), GV fresh 49.6% (254/512); *p* = 0.006) and 48 h in culture (GV vitrified 74.3% (310/417), GV fresh 62.7% (321/512); *p* < 0.0001: Table 1).

The IVM medium appeared to influence the MR, with higher values always evident in vitrified oocytes, although significant differences were only evident in the GVBD (GV vitrified 89.6% (240/268), GV fresh 78.2% (229/293); *p* < 0.0001), and the IVM 48 h (GV vitrified 82.5% (221/268); GV fresh 74.1% (217/293), *p* = 0.016) when the oocytes were cultured in CM2 medium (Table 2).

Overall, the percentage of oocytes that matured during the first 24 h was 54.93% (552/1005), which increased by 13.33% (134/1005) after 48 h to give a final proportion of 68.26% (686/1005) of oocytes that matured.

### 2.3. Activation Rate (AR)

The AR neither presented significant differences between the different groups studied (GV-Vit 46.9% (113/241), MII-Vit 53.7% (95/177), and Not-Vit 36.0% (9/25)), nor by the maturation medium used (CM1 53.6% (74/138) vs. CM2 46.9% (143/305)). However, the point of vitrification did appear to significantly affect the AR when the oocytes were matured in CM2 medium (MII-Vit 56.7% (59/104), GV-Vit 42.6% (75/176), Not-Vit 36.0% (9/25), *p* = 0.0026 and 0.0037), indicating that vitrification at the MII stage favors subsequent activation in this medium (Table 3 and Table 4).

### 2.4. Development Rate

The CRs were not significantly different between the three experimental groups, regardless of the culture medium used (GV-Vit 72.6% (82/113), MII-Vit 78.9% (75/95), and Not-Vit 77.8% (7/9)). Moreover, no significant differences were seen when we evaluated the average CN of the embryo on day 3 (Not-Vit 5.11 ± 2.892, GV-Vit 4.30 ± 2.741, and MII-Vit 4.89 ± 2.930). Taking into account the culture medium used and the group studied, we found that the CR after 48 h in culture in CM1 was significantly lower in oocytes that were vitrified at GV (60.5% (23/38)), as opposed to MII (88.9% (32/36), *p* = 0.007) (Table 3). However, no significant differences were observed between the groups when we evaluated the average CN in the embryos three days after activation (GV-Vit 3.97 ± 3.080, MII-Vit 4.47 ± 2.559). By contrast, in CM2, neither the CR (GV-Vit 78.7% (59/75), MII-Vit 72.9% (43/59), Not-Vit 77.8% (7/9)) (Table 4) nor the average CN of the embryo three days after activation (GV-Vit 4.30 ± 2.741, MII-Vit 4.89 ± 2.930, and Not-Vit 5.11 ± 2.892) were significantly different between the three groups.

Finally, there were no differences in the BR between the groups studied (GV-Vit 7.1% (8/113), MII-Vit 4.2% (4/95), and Not-Vit 77.8% (7/9)), and no significant differences were found in relation to the maturation media used (Table 3 and Table 4): CM1 (GV-Vit 7.9% (3/38) vs. MII-Vit 2.8% (1/36)); CM2 (GV-Vit 6.7% (5/75), MII-Vit 5.1% (3/59), Not-Vit 11.1% (1/9)).

## 3. Discussion

In this study, we focused on the effects of the timing of vitrification and of a specific IVM culture medium on the health and development of oocytes to be used in IVF procedures. Significantly higher MRs are obtained after 24 h and 48 h from vitrified GVs, as opposed to fresh oocytes, with significantly improved nuclear envelope breakdown again in vitrified oocytes. Conversely, and in terms of the stage of maturation at which the oocytes were vitrified (GV or MII), similar SRs and BRs were obtained, indicating that vitrification of GVs and their subsequent maturation seems a valid strategy to maximize the success of IVF/ICSI cycles.

Our SRs are similar to the those obtained elsewhere [14,34] and as with both groups of vitrified oocytes from IVF/ICSI cycles, the maturation stage (GV/MII) did not significantly influence the survival of vitrified oocytes. Likewise, our SRs are similar to those obtained in studies on GVs from unstimulated cycles [34,35,36]. Together, these SRs suggest that vitrification is a technique that can be applied to both immature and in vitro matured oocytes; moreover, they suggest that GVs recovered after stimulation or in a natural cycle have a comparable SR.

Culture conditions can alter the number of some oocyte structures during vitrification. Specifically, a decrease in the number of aquaporins has been described in oocytes matured in vitro, as opposed to in vivo, which may decrease the permeability of the membrane and augment their sensitivity to cryopreservation [37]. Nevertheless, the SR of MII oocytes obtained after IVM did not differ here from that of fresh oocytes, irrespective of the IVM medium used or the time in culture. Hence, neither the culture conditions nor the IVM times used here seem to affect the resistance to cryopreservation of the MII oocytes obtained.

In terms of IVM, the higher MR of vitrified GV relative to fresh oocytes has not been universally reported, although a higher MR after freezing GVs has been seen previously [38]. Elsewhere, similar MRs were reported between vitrified and fresh oocytes [29,39,40,41] or they were higher in non-vitrified oocytes [40,42]. Nevertheless, a recent meta-analysis questions the negative effects in vitrified GVs [15]. In fact, Ca^2+^ currents are necessary for rupture of the nuclear envelope and for the resumption of meiosis during cytoplasmic maturation [43]; in addition, an increase in intracellular Ca^2+^ was seen to be caused by the reagents used for vitrification, which aided the maturation of oocytes that had been previously vitrified [44,45,46,47]. The limited exposure to dimethylsulfoxide (DMSO) in the vitrification medium at room temperature may also improve the MR, without inducing spontaneous parthenogenesis [48]. Furthermore, vitrification of these oocytes in mammals appears to reduce in the intra-oocyte cAMP, favoring the resumption of meiosis and, hence, oocyte maturation [40]. These findings may explain the enhanced MR of GV-Vit oocytes detected here. Nevertheless, it must be noted that when the IVF medium was used, there were no significant differences in the MR of GVs, irrespective of whether they were vitrified prior to their IVM. Together, these data suggest that the vitrification and IVM conditions may influence the MR of oocytes, despite the ongoing controversy regarding the possible reasons underlying such an effect.

The use of an embryo culture medium designed to reach the blastocyst stage as a basal medium for IVM has previously been proposed, such as “Blastocyst culture medium” (BMI, Suwon, South Korea), “Blastocyst medium” (COOK Medical, Bloomington, Indiana), and CCM^TM^ (Vitrolife^®^, Gothenburg, Sweden [22,23,24]). Here, we obtained higher MRs with GVBD and 48 h oocytes using CCM^TM^ supplemented with hMG and SSS than when an early-stage gamete/embryo culture medium was used. With this medium, our 48 h MR was similar to those previously obtained with human oocytes from stimulated patients or from unstimulated cycles [23,24]. Embryo culture media designed for blastocysts simulate the micro-environment found in the uterus endometrium, and they provide the embryo with important energetic metabolites from the glycolytic pathway such as pyruvate and adenosyltriphospate (ATP). Such media has been supplemented with hMG, SSS, pyruvate, streptomycin, and penicillin [22], or only with hMG [24]. The presence of serum in the medium was seen to significantly improve the competence of immature oocytes to reach the MII stage, reducing the cytoplasmic damage induced by both vitrification and the removal of the granulosa cells [42]. This was sustained in other studies where the absence of granulosa cells and exposure to gonadotropins accelerates in vitro meiotic maturation [49,50,51]. Nevertheless, this acceleration may disrupt the synchronization between nuclear and cytoplasmic maturation, resulting in less competent oocytes and, thus, embryos with diminished developmental potential [40]. Our results confirm that the use of commercial media to blastocyst for IVM is reasonable, as long as they are properly supplemented. Furthermore, it seems that using immature oocytes obtained from natural [22,24] or stimulated [52,53] cycles does not influence the MR, even if the latter are no longer associated with the granulosa cells.

Only when culture medium to blastocyst supplemented with hMG and SSS was used, was a significantly higher AR evident when oocytes were vitrified after their maturation (MII-Vit). Our AR was lower than expected [54,55], which could be due to the modifications to the different protocols introduced. For example, we maintained the oocytes at 37 °C during the two activation steps instead of decreasing this to room temperature during ionomycin exposure [54]. Alternatively, the concentration of ionomycin was reduced by 50% in another protocol as electrical activation was performed prior to chemical activation [55]. Our data suggest that at least in the supplemented medium, the stage at which oocytes are cryopreserved may influence their AR. Activation depends on the ability of the oocyte to release Ca^2+^ in the presence of an adequate stimulus, as develops during maturation. For this, it is necessary to capacitate the oocytes during maturation through their sensitivity to inositol triphosphate (IP_3_), leading to a reorganization of the Ca^2+^ stores, an increase in their IP_3_ receptors, and a reorganization of the endoplasmic reticulum (ER). Cryopreservation studies on mouse oocytes concluded that warmed GVs can release Ca^2+^ in response to IP_3_, indicating that their membranes had not been damaged [41]. However, it seems that this sensitivity to IP_3_ does not exist in human oocytes [56]. This data can be explained by the functionality of the ER, an essential component of the Ca^2+^ release system that also acts as a store for this cation. This cytoplasmic structure remains intact after vitrified GV warming; yet, during their IVM, the expected reorganization does not occur and consequently, there is a decrease in the number of saccules and cortical aggregates [26,41,56].

Regarding the subsequent embryonic development, the excision rates that we obtained ranged from 60.5% to 88.9%, and day 3 embryos had between 4 and 5 cells on average, consistent with data from previous studies in which slower rates of division were seen in embryos that underwent IVM [6,57]. This slower development was attributed to defects in oocyte cytoplasmic maturation due to the loss of cytoplasmic proteins; and/or a decrease in the synchrony between nuclear and cytoplasmic maturation, as reflected by the microtubule dynamics and chromatin phosphorylation in IVM oocytes [58,59]. However, the same development potential to blastocyst was reported for oocytes matured both in vitro and in vivo, although only if they had not been preserved [60]. Nevertheless, if oocytes were vitrified, the main problem for their development was the embryonic transition at day 3, which many failed to overcome [17,60]. We mainly observed this noxious effect in oocytes vitrified at the MII stage that were matured in the medium with worst results (IVF), reaching a significantly higher rate of division (88.9%), but with less blastocyst formation (2.8%). 

In this study, the development to blastocyst of activated MII oocytes matured in vitro did not differ significantly, with rates comparable to those reported in the literature [54,55,57]. Nevertheless, when these oocytes are fecundated by ICSI, the published BRs are generally higher, reaching 32.7% [6,22,36], indicating that the method of activation may affect embryo development. Moreover, inadequate or incomplete maturation may be the main problem, not only for initiating the mechanisms driven by fecundation, but also to overcome the activation of the embryonic genome that occurs after the division from six to eight cells in humans [61]. In this sense, it has been reported that the different composition of aquaporins in the plasma membrane affects morula cavitation and, hence, blastocele formation, as seen when comparing in vitro and in vivo matured MII oocytes [37,62].

To conclude, the survival and developmental rates of oocytes are independent of the specific IVM medium used and the maturation stage of the oocytes prior to vitrification (GV or MII). MRs may be affected by the stage of vitrification, the maturation medium, and the time of culture. This may explain the controversies in the literature and for this reason, optimized procedures are required for IVM technique to improve. Since the data from animal models cannot always be extrapolated to human populations, well-designed human clinical trials may represent the final push required to improve the options of IVM. Thus, it is recommended that more experience in IVM is acquired in all the IVF laboratories.

## 4. Materials and Methods

This was a prospective, randomized cohort study that was approved by the Institutional Review Board at the Hospital Universitario y Politécnico La Fe (Valencia, Spain) and on which 481 patients treated for IVF or fertility preservation were enrolled, aged between 18 and 40 years old. All the women included in the study were fully informed of the study’s goals and they gave their signed consent to donate the GVs collected from their intracytoplasmatic sperm microinjection (ICSI) cycles carried out in the human reproductive unit of the aforementioned hospital.

### 4.1. Experimental Desing

**Figure 1:**
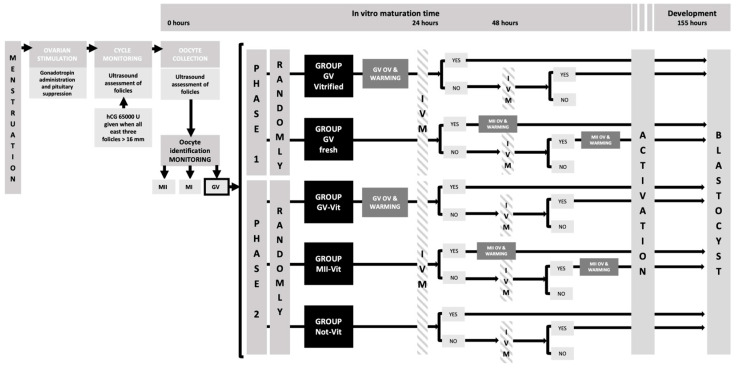
Experimental design describing the study groups and the methodology used, as well as the different in vitro maturation times (24 and 48 h): GV, prophase I; MI, metaphase I; MII, metaphase II; OV, oocytes vitrification; IVM, in vitro maturation.

To evaluate the effects of oocyte cryopreservation, and of IVM on activation and early embryonic development, two experimental phases were established in which different IVM culture mediums were tested: Phase 1, in which the CM1 medium was used for the preparation and handling of gametes, IVF, and embryo culture up to 2–8 cells (Universal IVF Medium, Origio^®^: Màlov, Denmark); and Phase 2, CM2 medium for culture from day 3 to the blastocyst stage and subsequent transfer (CCM^TM^: Vitrolife^®^, Gothenburg, Sweden), supplemented with human menopausal gonadotropin (hMG, Menopur^®^ 75 UI, Ferring^®^: Madrid, Spain) and synthetic serum substitute (SSS IrvineScientific^®^: Santa Ana, CA, USA). The different experimental groups established were: GV-Vit, GVs vitrified and then, matured in vitro; MII-Vit, MII oocytes vitrified after being matured in vitro; and Not-Vit, GVs matured in vitro, but not vitrified (Figure 1).

### 4.2. Oocyte Collection

All the participants underwent controlled ovarian stimulation following a short antagonist protocol. Pituitary suppression was achieved by administering rec-FSH (150–300 IU/day Gonal F 1050: Merck and Co, Madrid, Spain) and GnRH (Orgalutran^®^: MSD and Co., Hoddesdon, UK). When at least three follicles had grown to >16 mm, ovulation was induced by administering 250 μg of rec-hCG (Ovitrelle: Merck, London, UK). Oocyte retrieval was performed 36 h after hCG administration by ultrasound-guided transvaginal puncture–aspiration. Cumulus-corona-oocyte (CCO) complexes were denuded using hyaluronidase (SynVitro^®^ Hyadase, Origio^®^ Solution: Màlov, Denmark) for no more than 30 s with a denudation pipette (Denudation pipette Flexipet^®^: Cook^®^ Medical, Bloomington, IN, USA). Removal of the cumulus-corona cells is necessary to evaluate oocyte nuclear maturation. Despite coming from stimulated cycles, a total of 1113 GVs had an intracytoplasmic nucleus known as a germinal vesicle, characteristic of the prophase of the first meiotic division. In this study, we included all the oocytes that were relatively circular, between 120–140 μm in size, and with a homogeneous or slightly heterogeneous cytoplasm with no granularity due to inclusions or refractile bodies. We excluded 28 oocytes (2.5%) from the study as they were too large, presented dimorphisms in their zona pellucida, or had large vacuoles or signs of atresia/degeneration in their ooplasm.

### 4.3. In Vitro Maturation

GVs were cultured individually at 37 °C and 5% CO_2_ in micro-drops of culture medium (25 μL, CM1, or CM2) covered by mineral oil OVOIL^TM^ (Vitrolife^®^ Göteborg, Sweden). Oocytes were observed under an inverted microscope (Olympus, IX70, Tokyo, Japan) 20, 24, 44, and 48 h after the IVM commenced. Mature oocytes were considered to be those in which GV rupture was observed and the first polar body (PB) was seen in the perivitelline space within the first 48 h of culture.

### 4.4. Oocyte Vitrification and Warming

Vitrification was achieved in a vitrification/warming medium with the Cryotop^®^ open system device (Kitazato^®^: BioPharma Co., Shizuoka, Japan) and following a modified version of the protocol developed by Wang and Kuwayama [63,64]. The modification consisted of reducing the volumes indicated by the commercial company of each of the solutions used to tenth, maintaining the same exposure times to cryoprotectants. This protocol was employed with all the oocytes involved in this study, regardless of their maturation stage (GV or MII). The SR was evaluated microscopically with a Hoffman contrast 2 h after heating and it was based on observations of the previously described morphology, paying special attention to the integrity of the oocyte membrane.

### 4.5. Parthenogenetic Activation and Embryonic Development

**Figure 2:**
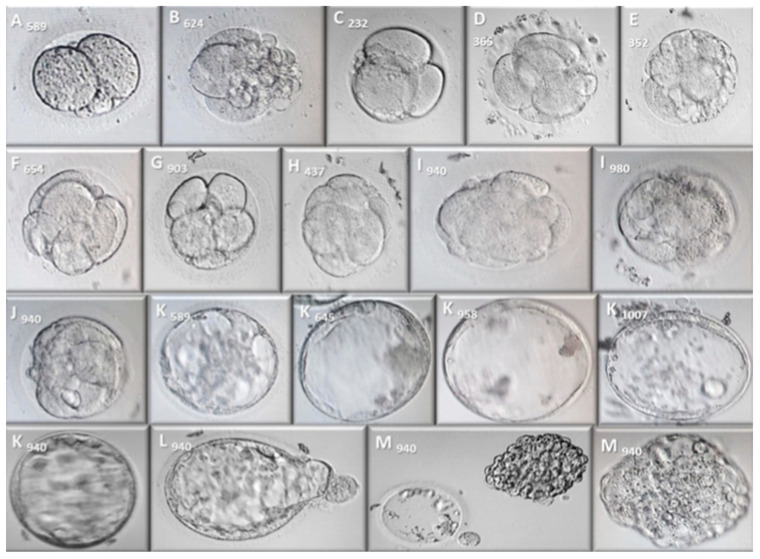
Bright field micrographs of embryos after activation. micrographs are labeled with a letter and a number. The letter refers to the stage of embryonic development and the number to the oocyte PI of the study. (**A**) 2-cell embryo. (**B**) 3-cell embryo. (**C**) 4-cell embryo. (**D**) 5-cell embryo. (**E**) 6-cell embryo. (**F**) 7-cell embryo. (**G**) 9-cell embryo. (**H**) Early compaction. (**I**) Morula. (**J**) Formation of the blastocyst cavity. (**K**) Fully expanded blastocyst. (**L**) Hatching blastocyst. (**M**) Hatched blastocyst. All micrographs were taken 20×, except the first image “M940” with a 10× objective.

Parthenogenetic activation (PA) of the oocytes was performed following the protocol developed by Paffoni, with minor modifications [57]. Oocytes were exposed to ionomycin calcium (10 μM: Sigma-Aldrich SRl, Milan, Italy) in an IVF medium for 5 min at 37 °C and 6% CO_2_ in the dark, and they were then exposed for 3 h to 6-Dimethylaminopurine (6-DMAP, 2 mM: Sigma-Aldrich SRl, Milan, Italy) in an IVF medium under the same conditions. The oocytes were subsequently cultured in micro-drops of G1^TM^Plus (25 μL: Vitrolife^®^, Frölunda, Sweden) supplemented with 10% SSS and overlayed with mineral oil. After 18–20 h, the oocytes that did not extrude the second PB and that had only one big pronucleus (PN) were considered activated. Parthenogenetic zygotes were subjected to sequential culture in G1^TM^Plus supplemented with SSS until day 3 post-activation and then, in CCM^TM^ supplemented with SSS until day 6 post-activation. Their developmental stage was evaluated every 24 h by observation under an inverted microscope for the 6 days in culture (Figure 2).

### 4.6. Statistical Analysis

The sample size required to detect a minimum of 35% difference in the oocytes between the control group (expected rate 15%) and any of the other experimental groups was calculated, obtaining a 95% confidence level (α = 5%) and a statistical power of 80% (β = 20%). The homogeneity of the groups was evaluated with the Kolmogorov–Smirnov test, and the differences between the quantitative variables were verified using a T-test or a Mann–Whitney U test. For the qualitative variables, the X^2^ test or Fisher’s test was used if both variables were dichotomous or some cells contained an expected frequency less than 5%. The different parameters were compared using contingency tables and X^2^ tests, with a level of α equal to 0.05: the maturation rates [MR = (MII oocytes (24–48 h)/GV oocytes) × 100], survival rate [SR = (viable devitrified oocytes/vitrified oocytes) × 100], activation rates [AR = (oocytes with 1PB/oocytes exposed to ICa^2+^+ 6-DMAP) × 100], cleavage rate [CR = (embryos dividing post-activation/zygotes 1PB) × 100]; blastocyst rate [BR = (cavitated embryos/zygotes 1PB) × 100], and embryo cell number at day 3 (CN = embryo cells number/zygotes). Differences were considered significant when *p* < 0.05.

## Figures and Tables

**Table 1:** Maturation rates (MR) evaluated relative to the maturation stage prior to vitrification in the groups studied (GV vitrified and GV fresh). MR in relation to germinal vesicle breakdown (GVBD) after 24 h (24 h) and 48 h (48 h) in culture. Average percentage by group and *p*-value.

GV Stage	MR		
	GVBD	24 h	48 h
Vitrified	83.7% (349/417)	59.2% (247/417)	74.3% (310/417)
Fresh	73.0% (374/512)	49.6% (234/512)	62.7% (321/512)
*p*-value	<0.0001	<0.0001	<0.0001

**Table 2:** Maturation rates (MRs) evaluated in relation to the maturation medium used for each of the groups studied: GV vitrified and GV fresh. The MRs in relation to germinal vesicle breakdown (GVBD) after 24 h (24 h) and 48 h (48 h) in culture. CM1, IVF medium; CM2, CCM supplemented medium. Average percentages relative to the groups and *p*-value.

IVM Medium	GV Stage	MR		
		GVBD	24 h	48 h
CM1	Vitrified	73.2% (109/149)	42.3% (63/149)	59.7% (89/149)
	Fresh	70.2% (207/295)	39.7% (117/295)	53.9% (159/295)
*p*-value		0.579	0.610	0.266
CM2	Vitrified	89.6% (240/268)	68.7% (184/268)	82.5% (221/268)
	Fresh	78.2% (229/293)	64.2% (188/293)	74.1% (217/293)
*p*-value		<0.0001	0.239	0.016

**Table 3:** Activation rate (AR), cleavage rate (CR), and blastocyst rate (BR) evaluated relative to the maturation stage prior to vitrification in the groups studied (GV and MII), both in CM1 medium. Average proportions by group and *p*-value.

CM1			
Vitrification Stage	RATES		
	AR	CR	BR
GV	58.5% (38/65)	60.5% (23/38)	7.9% (3/38)
MII	49.3% (36/73)	88.9% (32/36)	2.8% (1/36)
*p*-value	0.309	0.007	0.615

**Table 4:** Activation rate (AR), cleavage rate (CR), and blastocyst rate (BR) evaluated relative to the maturation stage prior to vitrification in the groups studied (GV-Vit, MII-Vit, and Not-Vit), both in CM2 (CCM supplemented medium). Average proportions by group and *p*-value. SD^a^ (MII-Vit vs. Not-Vit, *p*-value= 0.037) and SD^b^ (GV-Vit vs. MII-Vit, *p*-value= 0.026).

CM2			
Study Group	RATES		
	AR	CR	BR
GV-Vit	42.6% (75/176)	78.7% (59/75)	6.7% (5/75)
MII-Vit	56.7% (59/104)	72.9% (43/59)	5.1% (3/59)
Not-Vit	36% (9/25)	77.8% (7/9)	11.1% (1/9)
*p*-value	SD^ab^	NoSD	NoSD

## Data Availability

The data presented in this study are available in the article.

## Abbrevations

6-DMAP	6-Dimethylaminopurine
AR	Activation rate
ART	Assisted reproductive technique
ATP	Adenosyltriphospate
BR	Blastocyst rate
CM	Culture medium
CN	Embryo cell number at day 3
CCO	Cumulus-corona-oocyte
CR	Cleavage rate
ER	Endoplasmic reticulum
FSH	Follicle stimulating hormone
GnRH	Gonadotropin releasing hormone
GV	Germinal vesicle
GVBD	Germinal vesicle breakdown
h	Hours
hMG	Human menopausal gonadotropin
HTF	Human tubal fluid
hCG	Human chorionic gonadotropin
ICSI	Intracytoplasmic sperm microinjection
IP_3_	Inositol triphosphate
IVF	In vitro fertilization
IVM	In vitro maturation
MII	Metaphase II
MR	Maturation rate
OV	Oocyte vitrification
PA	Parthenogenetic activation
PB	Polar body
PN	Pronucleus
SR	Survival rate
SSS	Synthetic serum substitute
